# The ‘Schools Don’t Waste’ Program: A Theory-Informed Participatory Intervention to Reduce Plate Waste in Public School Canteens

**DOI:** 10.3390/nu18060885

**Published:** 2026-03-11

**Authors:** Mariusz Jaworski, Ewa Chojnowska

**Affiliations:** 1 Department of Education and Research in Health Sciences, Faculty of Health Science, Medical University of Warsaw, 00-581 Warsaw, Poland; 2 The Schools Don’t Waste (SDW) Project Team, 00-412 Warsaw, Poland; ewachojnowska78@gmail.com

**Keywords:** food waste, school canteens, intervention, behavioral economics, nutrition education, sustainability

## Abstract

Background/Objectives: Food waste in school canteens constitutes a significant environmental, organizational, and public health challenge. Despite numerous initiatives aimed at reducing plate waste, limited evidence exists on participatory interventions grounded in coherent theoretical frameworks and implemented in real school settings. This study aimed to evaluate the effects of the Schools Don’t Waste (SDW) program, a participatory, educational, and behavioral intervention based on the Needs-Based, Learner-Centered, Behaviorally Focused (NLB) model, in reducing visually assessed plate waste in primary school canteens. Methods: A quasi-experimental pre-post design without randomization was conducted in 37 public primary schools in Warsaw during the 2024/2025 school year. The intervention consisted of four stages: baseline plate waste assessment (T1), participatory roundtable meetings (T2), implementation of educational and organizational actions (T3), and post-intervention evaluation (T4). Plate waste was assessed using a standardized five-point visual scale. Differences between T1 and T4 were analyzed at the school level using the Wilcoxon signed-rank test. Implementation fidelity and its association with food waste reduction were explored using Spearman correlations. Results: A total of 4988 meals were assessed at baseline and 4080 at follow-up. Significant reductions were observed in the proportion of completely uneaten meals (Δ = −6.10 pp; *p* < 0.001; r = −0.67), meals with three-quarters uneaten (Δ = −5.76; *p* < 0.001), and meals with half uneaten (Δ = −7.97; *p* = 0.002). Overall uneaten meals decreased significantly (*p* = 0.004). Sixty-two percent of schools demonstrated measurable improvement, although fidelity indicators were not significantly correlated with outcomes. Conclusions: Participatory, low-cost interventions integrating educational and organizational components may effectively reduce plate waste in school settings, while structural and contextual factors appear to moderate intervention effectiveness.

## 1. Introduction

Food waste represents one of the key challenges facing contemporary food systems, carrying substantial environmental, economic, and health-related consequences. It is estimated that losses affect between 12% and as much as 40% of meals prepared in collective catering settings, including schools. For example, a study conducted across 78 primary schools demonstrated that as many as 28.6% of meals were not consumed. These data clearly indicate an urgent need for the implementation of effective preventive measures [1]. Similar trends have been observed in other analyses, where the average proportion of discarded and uneaten lunch meals ranged from 20% to 29%, depending on children’s age and the type of menu served [2]. Recent evidence from the United States further confirms the global nature of this phenomenon. A study conducted in 134 schools across 24 states reported that, on average, approximately 20% of prepared lunch meals were wasted. Losses were particularly high in primary schools and in settings where lunch breaks were short or scheduled early in the day [3].

The scale of food waste observed in schools has not only economic and health implications but also a significant environmental impact. It is estimated that wasting 20–29% of meals accounts for 14–18% of the global warming potential (GWP) generated by this sector [2]. At the global level, this problem is responsible for 8–10% of total greenhouse gas emissions, positioning food waste as the third-largest source of such emissions worldwide [4]. Adopting the higher-end estimates leads to even more alarming environmental implications. For example, if up to 40% of school lunch meals remain uneaten, this would imply a substantial amplification of resource loss, greenhouse gas emissions, and nutrient waste within institutional food systems. This reflects not only a substantial loss of natural resources but also considerable losses of nutrients, which ultimately reduce the effectiveness of collective catering programs whose primary objective is to support the health and proper development of children and adolescents.

Given the substantial environmental and health consequences, an increasing number of educational institutions are undertaking actions aimed at reducing food waste. However, there is currently no single universal and fully effective method for addressing this phenomenon. A narrative review by Gardner et al. [5] identified four primary directions of school-based interventions: (i) the introduction of more sustainable menus (including reduced meat provision, increased plant-based meals, and the use of local products), (ii) organizational and educational measures aimed at reducing food waste, (iii) the establishment of school gardens, and (iv) the expansion of nutrition education to include an environmental component. Previous analyses suggest that the implementation of these strategies may reduce greenhouse gas emissions by as much as 40–50% [1,2,3]. It should be noted that the effectiveness of these interventions has most often been evaluated using simple pre-post designs without control groups, which limits the reliability and generalizability of the reported findings.

The effectiveness of the aforementioned intervention strategies can be enhanced by complementing them with tools derived from behavioral economics (nudging). This approach draws on principles such as choice architecture, framing, and default effects, which aim to facilitate healthier and more sustainable choices without coercion. A systematic review demonstrated that measures such as modifying buffet layouts, using attractive dish names, serving fruits and vegetables in smaller, bite-sized portions, and increasing menu variety can effectively promote the selection of plant-based foods [6]. It should be emphasized that these interventions do not always translate into an actual increase in consumption and, in some cases, may even exacerbate food waste. This issue is particularly evident for plant-based meals, whose acceptance among students remains limited, as confirmed by previous analyses [2]. Therefore, the implementation of such measures should be preceded by informational and educational activities. Furthermore, experiences from the United States indicate that relatively simple organizational solutions can be highly effective. For example, the installation of milk dispensers reduced milk waste by 76%, and the introduction of school food-sharing stations significantly decreased overall food waste. In contrast, increasing students’ autonomy in selecting meal components (the so-called “offer versus serve” system) did not consistently produce the expected outcomes [3]. An important limitation of many of the aforementioned studies is the lack of systematic monitoring of implementation and the failure to account for the broader organizational context, such as the duration and structure of lunch breaks, which substantially limits the applicability and generalizability of the findings.

The analyses presented above indicate that, despite numerous attempts to implement interventions in schools, existing efforts have been characterized by limited sustainability, a low degree of standardization, and insufficient methodological rigor. In particular, there is a lack of studies grounded in coherent theoretical frameworks and involving the entire school community rather than only selected groups. In this context, the NLB approach (needs-based, learner-centered, behaviorally focused) described by Contento [7] appears especially relevant. This framework assumes that an effective intervention should: (i) be based on the actual needs of the community (needs-based), (ii) incorporate the active participation of all members of the school community (learner-centered), understood more broadly than solely in relation to students, and (iii) be oriented toward the modification of specific behaviors (behaviorally focused). Interventions designed in this way may enhance the sustainability of outcomes, support reductions in food waste, and simultaneously promote the development of pro-health and pro-environmental dietary habits throughout the school environment.

The NLB approach has most commonly been applied in the context of nutrition education, where it focuses on supporting learning processes and shaping individual pro-health attitudes. However, its potential for engaging entire school communities in activities aimed at reducing food waste has not yet been evaluated. In contrast to previous school-based interventions that have primarily evaluated isolated environmental or educational components, the present study conceptualizes the NLB framework as a structured implementation architecture rather than solely as an educational model. Specifically, the intervention was designed as an iterative cycle integrating needs-based diagnosis, stakeholder co-design, context-sensitive implementation, and behavioral feedback. By articulating participatory engagement as a mechanism of change, rather than merely as a delivery format, the study seeks to clarify how multi-component school interventions may generate organizational and behavioral shifts within complex institutional environments.

There is a lack of empirical studies examining the effectiveness of this model in the context of the complex environmental and organizational processes occurring within schools. Nevertheless, given its structure, integrating needs assessment, participatory action, and a focus on the modification of specific behaviors, the NLB approach appears to hold substantial potential for effectively reducing food waste in school settings. To address this gap, the “Schools Don’t Waste” program was developed and implemented in primary schools in Warsaw as a participatory educational and behavioral intervention aimed at achieving a sustained reduction in food waste. The program was based on the assumption that participatory planning, environmental restructuring, and behavioral feedback mechanisms would lead to reduced plate waste through enhanced awareness, shared responsibility, and improved meal satisfaction. According to the NLB framework, the combination of participatory engagement, behavioral feedback, and environmental restructuring was expected to reduce plate waste by strengthening students’ sense of ownership, food satisfaction, and collective responsibility within the school environment.

In the present study, the NLB framework is treated not merely as a design template for intervention development, but as a mechanism-informed implementation architecture in which participatory engagement is hypothesized to increase ownership and environmental alignment, thereby indirectly contributing to reductions in plate waste.

The primary objective of the study was to evaluate the effects of a theory-informed participatory implementation model (Schools Don’t Waste—SDW) on the reduction in food waste (plate waste) between baseline assessment (T1) and post-intervention evaluation (T4).

The secondary objective was to assess the extent to which the action plans developed during the roundtable meetings (T2) were implemented in practice during the educational and behavioral implementation phase (T3).

This intervention (SDW), based on the principles of the NLB approach [7], aimed not only to reduce food waste but also to strengthen pro-health and pro-environmental attitudes within the school community.

In relation to the primary and secondary objectives, the following research hypotheses were formulated:

**H1.**  *The visually assessed level of food waste (plate waste) will be significantly reduced between baseline measurement (T1) and final measurement (T4) following the implementation of the SDW intervention.*

**H2.**  *Schools that implement a greater proportion of the actions developed during the roundtable meetings (T2) will achieve greater visual reductions in food waste at T4.*

## 2. Materials and Methods

### 2.1. Study Design

The study employed a quasi-experimental pre-post design without randomization. The intervention was conducted in the canteens of selected public primary schools in Warsaw during the 2024/2025 school year (September 2024–June 2025). School recruitment lasted two weeks, from 1 to 15 September 2024. The implementation phase was carried out from October 2024 to May 2025, while the post-intervention evaluation (T4) was conducted in June 2025.

The project was based on the NLB approach [7] and comprised four main stages: (T1) visual assessment of food waste, (T2) roundtable meetings involving representatives of the school community, (T3) implementation of educational and behavioral activities, and (T4) evaluation.

The manuscript was prepared in accordance with the TREND (Transparent Reporting of Evaluations with Nonrandomized Designs) guidelines [8], and all required elements were incorporated into the study description. In addition, the intervention description and reporting were aligned with the TIDieR (Template for Intervention Description and Replication) checklist [9] to ensure completeness and replicability of the intervention details.

### 2.2. Sample Size and Participants

School recruitment for the study was conducted across all 18 districts of Warsaw. On 1 September 2024, an invitation to participate was sent to all public primary schools administered by the City of Warsaw (*n* = 223). The recruitment period lasted two weeks, during which schools could register by completing a registration form attached to the invitation. Fifty schools expressed willingness to participate, representing 22.42% of all invited institutions. Ultimately, 37 schools completed the project.

The inclusion criteria were as follows: (i) willingness to participate in a project lasting at least six months and involving the entire school community (school management, teachers, students, and canteen staff); (ii) participation of one class from grades IV, V, or VI; (iii) the presence of an operational school canteen providing lunch meals within a collective catering system; (iv) readiness of the school to set its own target for reducing food waste (e.g., a specified percentage reduction in plate waste during the school year); (v) the possibility of conducting observations in the canteen; (vi) the ability to collect questionnaire data from students; (vii) formal consent from the school principal; and (viii) a declaration of logistical support from school management.

Participation in the project was voluntary and was not associated with any financial incentives. Schools received certificates of participation. Schools that did not meet the inclusion criteria or withdrew before the baseline assessment were excluded from the analysis. In each participating school, one class with an average of 25 students took part in the study, resulting in approximately 925 students in total.

**Figure 1:**
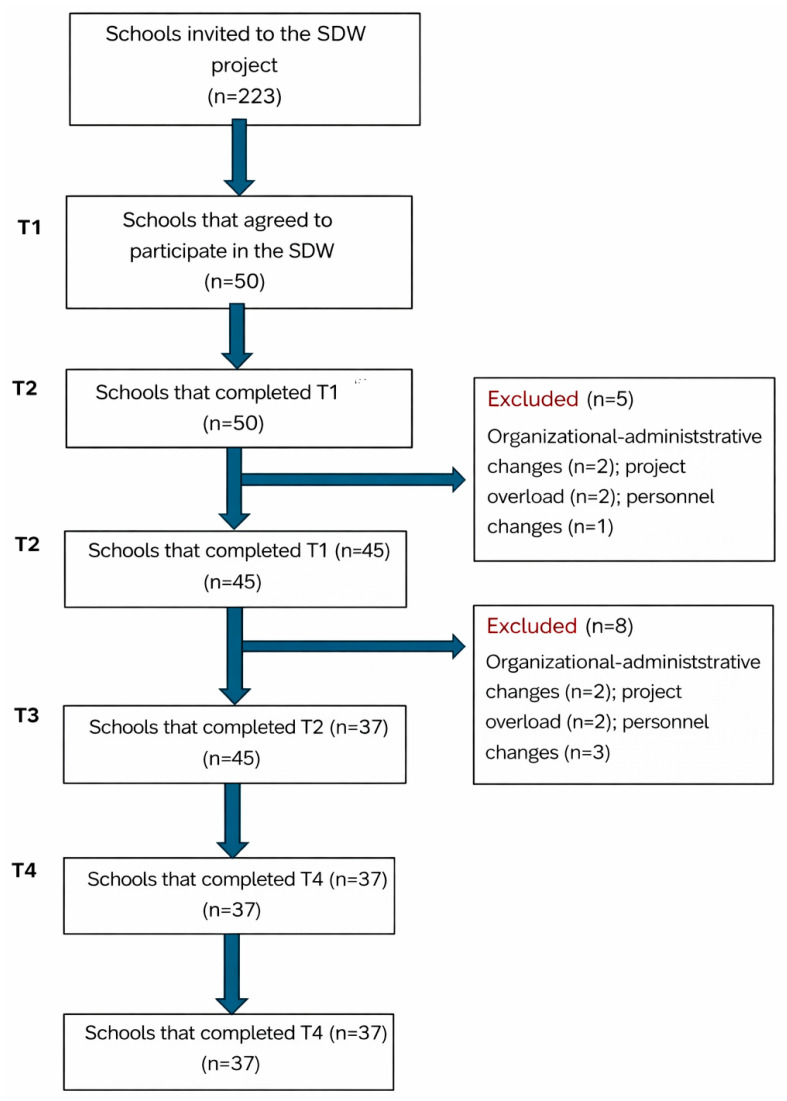
Participant Flow Diagram. *n*—number of schools; T1, T2, T3, T4—stages of the SDW (Schools Don’t Waste) project assessment.

Of the 223 public schools invited to participate, 50 (22.4%) expressed interest in joining the project. The baseline assessment (T1) was conducted in all 50 schools. During the course of the project, 13 schools withdrew before the implementation stage (T3), primarily due to organizational reasons (staff changes or interruptions in canteen operations). Ultimately, 37 schools completed the full cycle of intervention and evaluation (T4), representing 74% of the initially enrolled sample. Baseline structural characteristics of all initially enrolled schools (*n* = 50) are presented separately to describe the broader recruitment context. Complete T1 and T4 data were obtained for all 37 schools, enabling comparative analysis. The participant flow is presented in Figure 1 (Participant Flow Diagram).

Participation was voluntary at the institutional level. Invitations were distributed to all eligible public primary schools within the municipal system. However, 37 schools agreed to participate. The analytic dataset corresponds to this full participating sample, and no schools were excluded following baseline assessment.

To assess potential attrition bias, baseline characteristics of schools completing the intervention (*n* = 37) were compared with those withdrawing after baseline assessment (*n* = 13). No statistically significant differences were observed in structural catering characteristics (all *p* > 0.56) or baseline plate-waste indicators (% fully consumed and % fully uneaten meals; Mann–Whitney U = 243, *p* = 0.846). These findings suggest limited evidence of systematic attrition bias (Table 1).

The study had a pilot character and was aimed at assessing the feasibility and preliminary effects of the intervention under real-life school operating conditions. For this reason, no formal a priori sample size calculation was performed. The sample size was determined by the practical constraints of the municipal recruitment process (a two-week recruitment period) and the implementation capacity of the schools. A pragmatic objective was adopted, aiming to include a maximally heterogeneous sample of schools from all districts, with a minimum threshold of ≥30 schools considered sufficient to analyze contextual diversity and preliminary effects at the school level. No interim analyses or formal stopping rules were planned. The timeline and scope of implementation were determined by the school calendar and organizational arrangements with participating institutions.

### 2.3. Intervention Protocol

The intervention was entitled Schools Don’t Waste (Szkoła nie marnuje (in Polish—SDW) and is hereafter referred to as the SDW intervention. The intervention was developed based on the theoretical assumptions of the NLB approach (Needs-Based and Participatory Approach) [7], which emphasizes the importance of involving diverse representatives of the school community in the planning and implementation process. The foundation of this approach lies in the assumption that integrating the perspectives of the entire school community, students, teachers, canteen staff, parents, and school management, enables the development of goals that are feasible to implement and sustain in practice. As a result, the intervention not only addresses the actual needs of the school environment but also strengthens the community’s sense of shared responsibility for reducing food waste and promoting sustainable consumption patterns.

All intervention components were delivered in person at schools through interactive workshops, structured discussions, and participatory activities guided by project trainers.

All activities took place within school canteens and adjacent educational facilities (classrooms, assembly halls). All data were collected in school canteens and classrooms during regular school hours. Each participating school had an operational catering system, either in-house or provided by external contractors. All activities were conducted face-to-face within the school environment. No online or remote components were used. Each school constituted one intervention unit, and activities were conducted at the class level (one selected class per school).

#### 2.3.1. Stage 1: Baseline Assessment of Food Waste (Pre-Intervention Measurement—T1)

The visual assessment of uneaten lunch meals (T1) was conducted in school canteens. This assessment involved a visual evaluation of food remaining on students’ plates. The measurements were performed by students participating in the project under the supervision of teachers (school coordinators) and SDW project trainers. Plate waste analysis was conducted using a visual, rather than quantitative, method. This approach facilitated greater student engagement in the process while simultaneously increasing awareness of sustainable consumption and food waste reduction.

**Figure 2:**
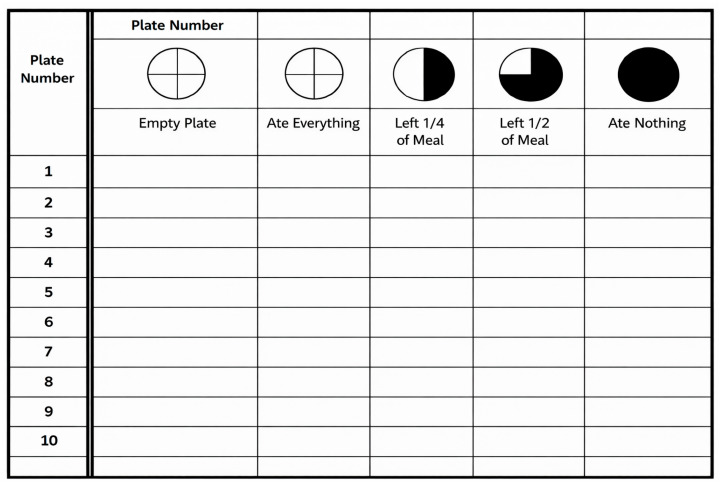
Baseline (T1) Plate Waste Observation Form.

The results were recorded using standardized observation forms, which were provided as Figure 2. The assessment was conducted over three consecutive days, during three lunch breaks in each school. In total, 4988 lunch meals were analyzed during the baseline assessment (T1). The diagnostic phase was carried out between 26 September 2024 and 30 June 2025.

#### 2.3.2. Stage 2: Roundtable Discussions (Measurement—T2)

In the second stage of the intervention, the “roundtable” method (T2) was applied to engage representatives of the entire school community in the process of planning actions aimed at reducing food waste. The discussions were based on the baseline assessment data (T1) and took the form of moderated workshop meetings.

The objectives of these meetings were: (i) to raise awareness among students, teachers, and kitchen staff about the scale of food waste in their school, (ii) to collaboratively identify solutions to reduce plate waste, and (iii) to define feasible changes that could be implemented within the school. Participants were encouraged to share their own observations and suggestions, all of which were documented and subsequently analyzed collectively in terms of feasibility. The meetings were conducted according to a structured scenario provided as Supplementary Material S1.

A total of 37 roundtable meetings were conducted, involving 239 participants (an average of eight participants per school). The meetings included students, teachers, school principals, parents, canteen staff, and project trainers. The method assumed equal voice among all participants, enabling open discussion and the joint development of priority strategies for reducing food waste. Stage T2 was carried out between 28 October 2024 and 15 April 2025.

#### 2.3.3. Stage 3: Implementation of Educational and Behavioral Changes (T3)

Based on the findings from T1 and the outcomes of the roundtable meetings (T2), each school implemented selected organizational and educational–behavioral actions (T3) tailored to its specific needs and organizational capacities. The intervention had a participatory character, meaning that the school community (students, teachers, canteen staff, and school management) decided on the choice of strategies and priority solutions.

Organizational actions included, among others: (i) improving the dining environment (esthetics, lighting, calm background music); (ii) reducing portion sizes with the option of additional servings; (iii) offering a choice between meal variants (e.g., two lunch sets); (iv) introducing salad bars, allowing students to decide on the type and quantity of vegetables; and (v) organizing tastings of new dishes.

Educational and behavioral actions included: (i) culinary workshops where students learned about food waste reduction and healthy eating principles; (ii) organizing School Health Days or Environmental Days incorporating food waste education; (iii) art and writing competitions promoting the idea of reducing food waste (e.g., for the best poster or slogan); (iv) engaging students as “project ambassadors” responsible for promoting good practices within the school community; and (v) incorporating children’s taste preferences into menu planning.

The implementation of changes occurred gradually in each school, with support from SDW project trainers. Each school had the autonomy to define its own food waste reduction goal (e.g., a specified percentage reduction in visually assessed plate waste during the school year). The average duration of this stage in individual schools was approximately six months, allowing for the observation of changes and their consolidation in everyday canteen practice.

The program was intentionally designed to allow for local adaptation; each school selected a context-adapted configuration of strategies within the shared NLB-guided implementation framework, based on baseline findings and institutional feasibility constraints. Although operational strategies varied across schools, all participating institutions were required to implement the four core stages of the NLB-guided intervention cycle: (1) baseline diagnostic assessment, (2) structured roundtable consultation involving stakeholders, (3) implementation of agreed changes, and (4) follow-up waste evaluation. This ensured structural comparability across sites while preserving contextual responsiveness as an intentional feature of the participatory design. The intervention lasted approximately six months in each school and comprised one baseline and one follow-up waste assessment, one 60 min roundtable session, and 4–6 months of educational and environmental activities (estimated 10–15 contact hours per school).

Each intervention was facilitated by trained project trainers (nutrition educators) with prior experience in school-based food waste programs. The trainers received a two-day standardized training in participatory facilitation, behavioral nudging, and data collection protocols.

#### 2.3.4. Stage 4: Evaluation (T4)

The evaluation of the intervention effects (T4) was conducted after the completion of the implemented organizational and educational–behavioral activities. Its purpose was to assess the effectiveness of the program in terms of the visual reduction in plate waste. Due to the nature of the intervention carried out in a school setting, blinding of participants and implementers was not feasible.

The evaluation included a visual assessment of plate waste in the school canteen (post-measurement—T4), conducted under the same conditions as the baseline assessment (T1) and using the same observation forms. Intervention fidelity was monitored through trainer checklists documenting the completion of each intervention stage (diagnosis, roundtable, implementation, evaluation). Adherence reports were submitted monthly by school coordinators.

The primary outcome was the level of food waste (plate waste), visually assessed at two measurement points: before the intervention (T1) and after its completion (T4), using a standardized observation form with a five-point scale (0 = no leftovers, 4 = entire portion left uneaten).

The secondary outcome was the extent to which the action plans developed during the roundtable meetings (T2–T3) were implemented.

All observations were conducted by trained evaluators using the same forms and assessment criteria at both measurement points, ensuring repeatability and reliability of the measurement.

In total, 4988 lunch meals (T1) and 4080 lunch meals (T4) were analyzed. A comparative analysis (T1 vs. T4) was then performed by juxtaposing the results from the baseline measurement with those from the post-intervention measurement. This enabled the assessment of the effectiveness of the actions implemented in each school. No modifications to the core intervention content or delivery were made during the study.

### 2.4. Primary and Secondary Outcomes

The primary outcome of the study was the change in the level of visually assessed plate waste between baseline (T1) and post-intervention evaluation (T4), measured using a standardized five-point observational scale (0 = no leftovers; 4 = entire portion left uneaten).

The secondary outcome was the level of implementation of the action plans developed during the roundtable meetings (T2), operationalized as the proportion of planned actions that were actually implemented during the educational and behavioral phase (T3) (implementation fidelity).

Additionally, associations between implementation fidelity and changes in food waste levels (Δ waste = T4 − T1) were explored.

### 2.5. Measurement Tool: Visual Plate Waste Assessment

Plate waste was assessed using a standardized visual observation form developed for the SDW project (Figure 2). The tool employed a five-point ordinal scale to estimate the amount of food left on students’ plates:

0—no leftovers,
1—approximately ¼ of the portion left,
2—approximately ½ of the portion left,
3—approximately ¾ of the portion left,
4—entire portion left uneaten.

The visual assessment method was selected due to its feasibility in real school conditions and its educational value, enabling active student involvement in the data collection process. Observations were conducted by trained students under the supervision of school coordinators and project trainers, following standardized instructions. Prior to data collection, project trainers provided standardized instructions and practical calibration exercises to ensure consistency in applying the five-point scale across schools. Although formal inter-rater reliability coefficients were not calculated, uniform training materials and supervision procedures were implemented to minimize variability in visual assessment.

Prior to baseline assessment, all evaluators (school coordinators and participating students) received standardized training conducted by project trainers. The training included (i) detailed explanation of the five-point scale, (ii) visual calibration using standardized example photographs of plates representing each waste category, and (iii) supervised pilot scoring of sample meals to ensure consistent interpretation of portion fractions. During site visits, project trainers conducted spot-check comparisons between evaluators to reinforce scoring consistency.

Although formal inter-rater reliability coefficients were not calculated due to the educational and participatory nature of the intervention, procedural standardization, supervised calibration, and structured observation forms were implemented to reduce subjective variability. This approach aligns with feasibility-oriented waste monitoring protocols used in school-based settings where weight-based auditing is impractical.

The same tool, criteria, and procedures were applied at both measurement points (T1 and T4), ensuring consistency and comparability of the data across time.

To further minimize observer bias, assessment was conducted using structured observation forms immediately after meal completion, reducing retrospective estimation. Project trainers periodically monitored adherence to scoring criteria during site visits to reinforce consistency across schools.

To enhance methodological transparency and procedural consistency across schools, standardized training materials, calibration exercises, structured observation forms, and supervisory spot-check procedures were implemented at both measurement points. These quality control procedures were designed to minimize subjective variability inherent in visual assessment methods.

### 2.6. Baseline Characteristics of Participating Schools and Catering Organization

Prior to the intervention, contextual data were collected regarding the organization of school catering in participating schools. These variables included: type of catering system (in-house kitchen vs. external provider), number and organization of lunch breaks, availability of menu information for students and parents, portioning practices, availability of second servings, presence of salad bars, and flexibility in portion size selection. These characteristics were considered important contextual factors potentially influencing plate waste levels and were therefore documented before the intervention.

### 2.7. Data Analysis

The unit of analysis was the school, treated as a coherent unit of intervention implementation and outcome measurement. Complete data across all measurement points (T1, T2, T3, and T4) were obtained from schools that participated in and completed the full intervention cycle. Therefore, no data imputation procedures were required. The mean level of food waste was calculated for each school at T1 and T4, and the differences between these values were subsequently compared at the school level (aggregated change score).

Individual plate observations were aggregated at the school level by calculating the mean waste score and proportional distribution of waste categories per school at T1 and T4. This aggregation approach was adopted because the intervention was implemented at the school level and contextual factors (canteen organization, staffing, lunch scheduling) operated at that level. As such, schools constituted the appropriate analytical unit, and given that the intervention operated at the institutional level, aggregation to the school level was conceptually consistent with the implementation unit. However, this approach does not allow modeling of within-school clustering effects.

Differences in food waste levels between T1 and T4 were analyzed using the Wilcoxon signed-rank test for paired samples (non-parametric data), and effect sizes were calculated (r = Z/√N) with 95% confidence intervals. Results for the primary endpoint (change in plate waste level from T1 to T4) are presented with 95% confidence intervals to allow assessment of estimate precision, which is recommended as an alternative to post hoc power analyses in pilot studies.

Additionally, the relationships between the number of planned and implemented actions and the change in food waste levels (Δ waste = T4 − T1) were analyzed using Spearman’s rank correlation coefficient. The implementation fidelity indicator was operationalized as the ratio of implemented to planned actions multiplied by 100. This indicator reflects the extent to which schools adhered to the intervention plan, providing a quantitative measure of its implementation in a real-world sustainability context.

Although individual plate observations were recorded at the student level, the intervention was implemented at the institutional level and organizational decisions were made at the school level. Therefore, aggregated school-level indicators were treated as the primary unit of analysis. Given the relatively small number of institutional units (*n* = 37), estimation of multilevel models including building-level covariates was considered statistically unstable and potentially overparameterized. Future studies with larger institutional samples may apply hierarchical modeling approaches.

In response to potential concerns regarding cluster-level confounding, an exploratory school-level linear regression analysis was conducted as a sensitivity analysis. Change in percentage of uneaten meals (T4 − T1) was entered as the dependent variable. Baseline waste level and selected structural characteristics (catering type, number of lunch breaks, salad bar availability, portion flexibility, and ability to request additional portions) were included as predictors. Variance inflation factors (VIF) were examined to assess multicollinearity.

Statistical analyses were performed using IBM SPSS Statistics for Windows, Version 14.0 (IBM Corp., Armonk, NY, USA). The interpretation of the results was limited to exploratory conclusions, taking into account potential systematic biases typical of non-randomized study designs.

### 2.8. Ethical Considerations

The project was conducted in accordance with the principles of the Declaration of Helsinki. The intervention was approved and supported by the Center for Social Communication of the City of Warsaw, which formally invited schools to participate in the Schools Don’t Waste program. The research team from the Medical University of Warsaw supervised the methodological and ethical aspects of the study to ensure compliance with scientific and ethical standards.

The project did not involve the collection of personal or health-related data and therefore did not require separate approval from a university bioethics committee. Participation was voluntary, and all schools provided written institutional consent prior to participation in the intervention.

## 3. Results

### 3.1. Baseline Characteristics of Participating Schools

Table 2 presents the baseline characteristics of all schools that participated in the initial diagnostic phase (T1) (*n* = 50), including the number of students using the canteen, district location, type of catering system (in-house kitchen vs. external provider), and selected organizational indicators. These data describe the broader baseline sample prior to attrition.

In the studied schools, meal provision was more commonly based on services provided by external catering operators, whereas meals prepared in on-site school kitchens were less frequent. The schedule of lunch breaks varied across schools. However, most schools offered two lunch breaks, while a smaller proportion provided three or more. In all schools, students and parents had prior access to the weekly menu, and meals were consistently portioned and served by canteen staff, without elements of self-service.

Only a small number of schools allowed students to choose between different portion sizes or operated salad bars. Similarly, the possibility of receiving additional servings was available only in some schools. Overall, the catering organization model demonstrated a relatively high level of uniformity, with limited flexibility regarding portion size and additional options (Table 2).

On average, each participating school had 620 students enrolled. Approximately 343 students used the school lunch service, representing a mean of 56.48% of the total student population. The study involved 16 fifth-grade classes, 15 seventh-grade classes, 5 sixth-grade classes, and one eighth-grade class, corresponding to students aged approximately 11–14 years within the Polish education system.

### 3.2. Primary Outcomes

#### 3.2.1. Stage 1: Baseline Assessment of Plate Waste

A total of 4988 lunch meals (T1) were included in the analysis. On average, 135 meals were assessed per school, of which only 46 were, on average, fully consumed. It was observed that a substantial proportion of lunch meals were not fully eaten by students. Only 34% of meals were consumed in their entirety, whereas in 66% of cases students left plate waste of varying amounts.

The most frequently observed situation was leaving approximately one-quarter of the portion uneaten (29% of all meals). Approximately 19% of meals were consumed only halfway, and in 12% of cases three-quarters of the portion remained uneaten. In extreme cases, 6% of meals were not consumed at all.

Analysis of data from 37 schools revealed considerable variability in meal consumption patterns. In only eight schools was a favorable ratio observed, where the number of fully consumed meals exceeded the number of uneaten meals. In the remaining schools, the pattern was unfavorable, with students more often leaving a substantial part of the meal or not consuming it at all.

#### 3.2.2. Stage 4: Post-Intervention Evaluation (T4)

A total of 4080 lunch meals were included in the post-intervention assessment (T4). On average, 110 meals were analyzed per school, of which 40.15% were fully consumed. This indicates an increase in the proportion of meals eaten in full compared with the baseline stage (T1).

The analysis of plate waste showed that students most frequently left approximately one-quarter of the portion uneaten (33.09% of all meals). Meals consumed halfway accounted for 15.83%, while in 9.85% of cases three-quarters of the portion remained uneaten. In extreme cases, 1.76% of meals were not consumed at all, which also represents an improvement compared with the baseline results.

As previously observed, considerable variability in consumption patterns remained across schools. In 11 schools, a favorable ratio was recorded, where the number of fully consumed meals exceeded the number of uneaten meals. In the remaining schools, students still more frequently left a substantial portion of their lunch uneaten.

#### 3.2.3. Comparison of Differences Between Baseline (T1) and Post-Intervention Evaluation (T4)

All 37 schools that initiated the project completed the full intervention cycle. Among the participating schools, 62% (*n* = 23) recorded a measurable reduction in food waste, whereas 12 schools experienced a deterioration in outcomes and two schools showed no change. On average, the proportion of meals consumed in full increased by approximately 4%, with some schools achieving an increase of up to 41%, indicating substantial variability in intervention effects.

Compared with the baseline stage (T1), the proportion of completely uneaten meals decreased by an average of 6.1 percentage points (Δ = −6.10; Z = −4.052; *p* < 0.001; r = −0.67), representing a large effect size. Similarly, significant reductions were observed in the categories “¾ of the portion left uneaten” (Δ = −5.76; Z = −3.397; *p* < 0.001; r = −0.56) and “½ of the portion left uneaten” (Δ = −7.97; Z = −3.164; *p* = 0.002; r = −0.52). The total number of uneaten meals also decreased significantly (Δ = −22.13; Z = −2.867; *p* = 0.004; r = −0.47).

Although the increase in the proportion of meals fully consumed (Δ = +4.01) did not reach statistical significance (Z = −0.778; *p* = 0.436; r = −0.13), a clear trend toward reducing the amount of plate waste was observed (Table 3).

To address potential cluster-level confounding, an exploratory school-level linear regression was conducted with change in percentage of uneaten meals (T4 − T1) as the dependent variable and baseline waste level plus structural characteristics (catering type, number of lunch breaks, salad bar availability, portion flexibility, and ability to request additional portions) as predictors. The model was statistically significant (F(6,29) = 4.94, *p* = 0.001, adjusted R^2^ = 0.403). Baseline waste level was a strong predictor of change (β = 0.60, *p* < 0.001). The strong association between baseline waste and change likely reflects both statistical regression to the mean and greater improvement potential in schools with initially higher waste levels. Among structural characteristics, only salad bar availability was associated with greater reduction in waste (β = −0.36, *p* = 0.021), whereas other variables were not significant predictors. Detailed regression coefficients are presented in Table 4. Given the small number of institutional units, regression results should be interpreted cautiously.

### 3.3. Secondary Outcomes

#### 3.3.1. Roundtable Discussions

In the second stage, 37 roundtable meetings were conducted. Each meeting lasted approximately 60 min and was moderated by *Schools Don’t Waste* project trainers. The structure of participants was diverse and reflected the multi-stakeholder nature of the participatory approach. In each school, a project coordinator was present (most often a teacher, school counselor, or class tutor) (*n* = 37; 100%), as well as student representatives—from selected classes (grades V–VII) and from the Student Council (*n* = 30; 81.1%).

In 51.3% of meetings, school principals or vice-principals participated (*n* = 19), and in 70.3% of meetings, representatives of Parents’ Councils were present (*n* = 26). In over 59% of schools, kitchen staff or representatives of the catering company attended the meetings (*n* = 22), allowing practical aspects related to menu planning and portioning to be addressed. The Schools Don’t Waste project trainers also supported the workshop process and ensured methodological consistency.

Each school was required to define its own food waste reduction goals. Of the 37 schools, 24 set one primary goal, while the remaining schools (*n* = 13) defined two goals. The most frequently indicated objective was to increase the proportion of meals consumed in full, declared by 31 schools (83.8%). On average, schools aimed to improve this indicator by approximately 10%; four schools set a target of 5%, and one school adopted a more ambitious target of 25%. Additionally, in 18 schools (48.6%), the goal was to reduce the number of meals not fully consumed.

Across all schools, a total of 156 informational and educational activities were planned (an average of four per school) and 154 actions directly related to canteen organization (an average of five per school). The meetings also served an educational function by increasing awareness among students and teachers regarding the scale of the problem, strengthening their sense of responsibility, and engaging them in the shared decision-making process concerning the direction of changes.

Across the 37 schools, a total of 159 actions aimed at reducing food waste were planned. The most frequent initiatives concerned organizational and logistical changes (35 actions), including adjustments to lunch break schedules, extending the time available for meal consumption, and reorganizing the canteen space.

An important area of intervention also involved modifications to portioning practices (29 actions), such as introducing smaller portions with the option of additional servings and providing separate plates for salads and fruits. Further actions focused on menu modification and improving meal quality (23 actions), as well as education and the promotion of healthy eating habits (25 actions), including workshops, competitions, and visual promotion of meals.

Measures encouraging students to try new dishes were introduced in 10 schools, while 12 schools decided to systematically assess students’ preferences using surveys and feedback systems. Food redistribution and reuse strategies were planned in 10 schools, and 15 actions addressed canteen infrastructure and resources (e.g., equipment purchase and improvement of technical conditions).

This wide range of initiatives reflects a multidimensional approach to reducing food waste within the school environment.

As part of the informational and educational component, a total of 156 initiatives were planned, focusing on a broad range of communication and awareness-raising activities. The most frequently implemented solution consisted of information campaigns directed at students and parents (43 actions), including posters, infographics, electronic messages, and reminders regarding meal choices.

An important element also included surveys and systematic monitoring of students’ preferences (31 actions), as well as educational lessons and workshops (22 actions) promoting healthy eating and pro-environmental attitudes. In 23 schools, competitions and creative activities were organized, while 15 institutions prepared assemblies, debates, and other community-oriented events.

The initiatives also incorporated modern communication formats, such as educational videos, multimedia presentations, and 3D posters (11 actions). Importantly, some of these activities involved the active participation of students and parents in decision-making processes related to the functioning of the school canteen (11 actions).

The comparative analysis revealed a significant decrease in the number of actions implemented relative to those initially planned. In the informational component, the median difference was Δ = −1.75 [IQR −3.30 to 0.00]; Wilcoxon test results: Z = −3.675; *p* < 0.001; r = −0.60, indicating a large effect size.

An even more pronounced decline was observed in the canteen-related component (Δ = −1.50 [IQR −3.00 to 0.00]; Z = −4.572; *p* < 0.001; r = −0.76), corresponding to a very large effect size. These findings confirm that a proportion of the planned actions was not fully implemented by the end of the intervention, particularly in areas requiring organizational changes within school canteens.

#### 3.3.2. Associations Between Implementation Fidelity and Changes in Food Waste

A correlation analysis was conducted to examine the relationships between the number of planned and implemented actions, the implementation fidelity indicator, and the change in food waste levels (Δ waste = T4 − T1). As presented in Table 5, none of the analyzed associations reached statistical significance (all *p* > 0.05). The number of planned actions was not significantly correlated with the change in food waste levels (ρ = −0.07; *p* = 0.70), and a similar pattern was observed for the number of implemented actions (ρ = 0.07; *p* = 0.66).

Although the association between implementation fidelity and waste reduction did not reach statistical significance (ρ = 0.20; *p* = 0.23), this result may indicate that quantitative adherence alone does not fully explain intervention effectiveness. In complex school environments, the qualitative configuration, contextual fit, and strategic coherence of implemented actions may be more influential than the sheer number of activities completed. This finding challenges the linear assumption that greater implementation volume automatically produces stronger outcomes and highlights the importance of context-sensitive alignment.

This weak, non-significant relationship may indicate that the effectiveness of the intervention was influenced by other contextual or behavioral factors (e.g., staff engagement, kitchen organization, or routines in meal serving), which is consistent with observations from real-world sustainability interventions conducted in educational settings.

This pattern suggests that not all implemented actions were functionally equivalent. Structural modifications directly affecting portioning or meal selection may exert a stronger influence on waste patterns than communication-based educational campaigns alone. Future studies should therefore differentiate between configuration types rather than relying solely on quantitative fidelity indicators.

## 4. Discussion

### 4.1. Summary of Main Findings

The aim of the SDW intervention was to reduce the visually assessed level of plate waste between the baseline measurement (T1) and the post-intervention evaluation (T4). Of the 37 schools that completed the full intervention cycle, 62% recorded a measurable improvement. Compared with T1, a significant reduction was observed in the proportion of completely uneaten meals (Δ = −6.10 percentage points; *p* < 0.001; r = −0.67), as well as in the proportion of meals where three-quarters or one-half of the portion remained uneaten (*p* < 0.01). The total number of uneaten meals also decreased significantly (*p* = 0.004). The effect sizes observed for reductions in high-waste categories were moderate to large (e.g., r = −0.67 for completely uneaten meals), indicating that the changes were not only statistically significant but also practically meaningful at the school level.

The magnitude of the observed effects varied substantially across schools, with some institutions demonstrating minimal change and others achieving reductions exceeding 30 percentage points in high-intensity waste categories. This dispersion indicates heterogeneous intervention responsiveness rather than a uniform treatment effect.

Exploratory inspection suggested that schools demonstrating deterioration were characterized by minimal structural modifications and unchanged lunch scheduling practices, although formal moderator analyses were beyond the statistical scope of the present sample.

At the same time, the increase in the proportion of meals consumed in full did not reach statistical significance. This pattern suggests that the intervention primarily reduced high-intensity waste (e.g., fully uneaten meals and large leftover proportions) rather than uniformly increasing complete consumption. From a practical perspective, reducing extreme waste categories may represent a more meaningful sustainability outcome than marginal increases in full meal completion rates. The results indicate substantial variability in intervention effects across schools, suggesting an important role of organizational and environmental factors. The association between the level of implementation of planned actions and the reduction in food waste was not statistically significant, although a positive trend in this relationship was observed.

These findings suggest that evaluating intervention success solely on the basis of full meal completion rates may underestimate meaningful sustainability gains occurring within intermediate waste categories.

### 4.2. Comparison with Previous Literature

Prior school-based food waste interventions indicate that multi-component strategies integrating environmental restructuring with participatory engagement produce more consistent reductions in plate waste than stand-alone educational approaches [10,11,12,13,14]. In particular, interventions combining student involvement in decision-making with structural modifications to the dining environment have reported measurable reductions in high-intensity waste categories [10,11].

Behaviorally informed environmental adjustments, including modifications in food presentation, portioning practices, serving structure, and choice architecture, have been associated with reduced discarding of fruits and vegetables, although reported effect magnitudes vary substantially across institutional contexts [12,13]. Flexible serving strategies and context-sensitive menu planning appear particularly relevant in reducing mismatches between portion size and appetite [14]. Programs embedding nutrition education within operational catering changes demonstrate greater sustainability than isolated educational campaigns [15,16].

At the same time, existing literature emphasizes that contextual conditions, such as lunch duration, infrastructure, portioning flexibility, and staffing capacity, may function as moderators of intervention effectiveness [5,16]. The variability observed in the present study aligns with this broader pattern, suggesting that environmental alignment and institutional feasibility play a central role in translating participatory planning into measurable reductions in waste.

Prior research suggests that environmental restructuring tends to yield more consistent reductions in high-waste categories than stand-alone educational components [12,13,14], whereas participatory engagement enhances sustainability and behavioral internalization when organizational feasibility is ensured [10,16]. The present findings are consistent with this trajectory, particularly in demonstrating stronger reductions in extreme leftover categories rather than uniform increases in complete meal consumption. In this sense, the study offers incremental empirical support for environmental alignment mechanisms while advancing the field by embedding these mechanisms within a structured NLB-informed participatory implementation cycle.

### 4.3. Interpretation of Mechanisms

The most favorable outcomes were observed in schools that introduced structural modifications such as salad bars, flexible portioning, and greater student autonomy in selecting meal components. Although the magnitude of change falls within ranges reported in prior school-based interventions [12,13,14], the pattern of reduction, particularly within extreme leftover categories, more closely resembles studies emphasizing structural adaptation rather than education alone.

Such practices likely reduce mismatch between portion size and individual appetite while simultaneously strengthening perceived agency in meal selection. Evidence from portion-adjustment interventions and self-service models suggests that aligning serving practices with individual needs reduces high-intensity waste more effectively than informational strategies alone [13,14].

From a behavioral economics perspective, expanding choice architecture may increase perceived control and meal acceptance, thereby decreasing rejection-driven waste. Interventions modifying product placement, accessibility, and self-service structures have been associated with measurable reductions in discarded food, particularly in fruits and vegetables [12,13]. Positioning students as active participants in shaping food provision appears to reinforce norm formation and responsibility, especially when embedded within structural changes to the dining environment [16].

Conversely, schools in which food waste increased largely did not implement substantive organizational modifications. This pattern suggests that educational initiatives operating in isolation may be insufficient when structural constraints remain unaddressed, a limitation repeatedly identified in prior evaluations of school-based nutrition programs [10].

Collectively, the pattern of results indicates that environmental alignment, organizational feasibility, and student engagement operate synergistically in shaping plate waste trajectories. Structural adaptations appear particularly relevant for reducing high-intensity waste categories, whereas isolated educational components may exert limited influence when contextual barriers persist.

The substantial variability across schools further indicates that the intervention functioned as a context-sensitive implementation process rather than as a uniform treatment effect. In settings where structural adaptations aligned with local constraints, measurable reductions were observed. Where logistical, temporal, or staffing limitations persisted, effects were attenuated. This heterogeneity suggests that contextual alignment may moderate effectiveness more strongly than implementation intensity alone.

### 4.4. Role of Participatory Approach and the NLB Model

A central premise of the Schools Don’t Waste (SDW) program was that reductions in plate waste require more than isolated educational messages or single environmental “nudges.” The intervention was therefore designed as a theory-informed participatory implementation process grounded in the Needs-Based, Learner-Centered, Behaviorally Focused (NLB) model [7]. While participatory and multi-stakeholder approaches have previously been recommended in school sustainability research [5,10,16], SDW applied the NLB framework not only as an educational philosophy but as an organizing logic linking diagnosis, co-design, and behaviorally targeted implementation.

Importantly, the absence of a statistically significant association between implementation fidelity and waste reduction (reported in Section 3.3.2) challenges a linear assumption that greater quantitative adherence automatically produces stronger outcomes. This finding suggests that the qualitative configuration, contextual fit, and strategic coherence of implemented actions may be more relevant than their number. In complex school environments, effectiveness may depend on alignment with local organizational dynamics rather than on implementation volume alone.

Within SDW, the needs-based component was operationalized through the baseline plate-waste assessment (T1), which provided each school with a context-specific diagnostic profile rather than relying on generalized assumptions about waste drivers. This approach aligns with recommendations that interventions be adapted to local organizational conditions [5]. The learner-centered component was implemented through structured roundtable meetings (T2) involving students, teachers, school management, parents, and canteen staff. Previous research has emphasized that multi-level engagement enhances sustainability and shared responsibility [5,10,16]. In SDW, however, stakeholder involvement was not limited to consultation; it structured the formulation of feasible, context-sensitive action plans. Unlike prior school-based interventions that primarily introduced environmental adjustments or educational components as discrete strategies, SDW operationalizes participatory engagement as a mediating mechanism rather than merely a delivery format. In this configuration, stakeholder involvement is conceptualized as generating ownership, contextual alignment, and behavioral salience, which in turn influence high-intensity waste behaviors. Thus, the theoretical contribution does not reside in the novelty of individual intervention components, but in their structured integration within a mechanism-informed implementation architecture grounded in the NLB framework.

The behaviorally focused dimension was reflected in the translation of roundtable conclusions into observable environmental and educational modifications during the implementation phase (T3). These included both structural strategies (e.g., portion flexibility, self-service elements, changes to dining conditions) and educational actions. Similar environmental modifications have been shown to influence food selection and waste patterns [12,13,14], and participatory models integrating environmental and educational components have been highlighted as promising in school-based programs [10,15]. However, SDW combined these elements within a coherent iterative cycle: diagnosis → co-design → implementation → feedback (T4), consistent with implementation science perspectives on complex interventions.

Importantly, SDW conceptualized participation as a mechanism of change rather than merely a delivery format. The intervention assumed that participatory co-design would increase perceived ownership of the canteen environment and strengthen shared responsibility for feasible modifications. This mechanism is consistent with findings suggesting that student engagement enhances both sustainability and behavioral internalization [10,16]. Through this pathway, improved alignment between meal provision and student preferences was expected to reduce the likelihood of substantial plate waste.

Two complementary processes may help explain the observed outcomes. First, environmental restructuring, such as flexible portioning or self-service salad bars, can reduce mismatch between appetite, time constraints, and portion size. Prior studies have reported that such structural adjustments reduce plate waste, particularly when aligned with student preferences [13,14]. Second, behavioral feedback and norm formation may emerge when students are directly involved in observing and discussing food waste. As suggested in previous interventions, positioning students as active contributors rather than passive recipients supports stronger internalization of pro-health and pro-environmental behaviors [10,16].

The empirical pattern observed in SDW is compatible with this mechanism-oriented interpretation. The most pronounced reductions occurred in categories representing larger amounts of leftovers (fully uneaten meals and meals with half or three-quarters uneaten), suggesting that the intervention primarily reduced extreme mismatches rather than uniformly increasing complete consumption. Similar variability has been observed in other school-based interventions, where environmental modifications yielded stronger effects on high-waste categories than on total meal completion rates [12,13,14].

At the same time, the heterogeneity of effects across schools indicates that participatory engagement alone does not guarantee uniform outcomes. SDW was intentionally designed to allow local adaptation, with schools selecting strategies based on contextual feasibility. While adaptability has been identified as a strength of school sustainability initiatives [5], it also implies that contextual moderators, such as canteen logistics, staffing, and lunch break organization, may influence the translation of action plans into measurable reductions in waste.

The absence of statistically significant correlations between quantitative fidelity indicators and waste reduction further supports a mechanism-focused interpretation. Rather than suggesting that implementation does not matter, this finding indicates that the type and contextual fit of implemented actions may be more important than the number of actions completed. Similar conclusions have been implied in prior research, where structural alignment and feasibility were more predictive of outcomes than intervention intensity alone [12,15]. Future analyses should therefore move beyond simple counts of activities and explore configuration-based approaches that distinguish between structural, educational, and contextually aligned strategies.

**Figure 3:**
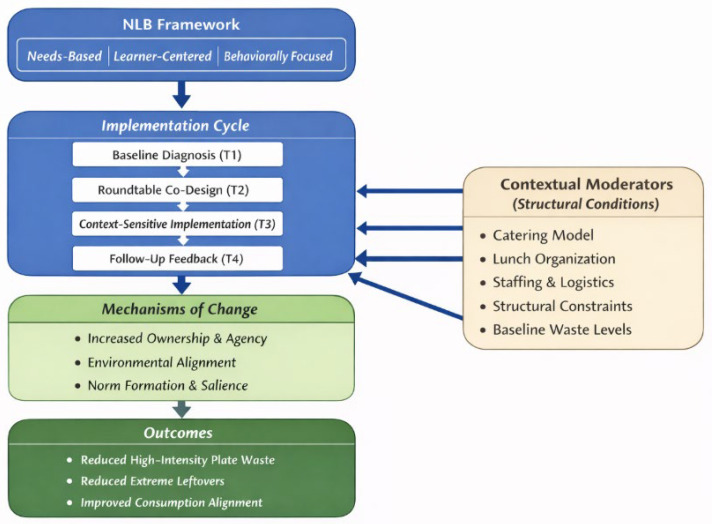
Conceptual model of the NLB-informed participatory mechanism in the SDW program. The model illustrates how the Needs-Based, Learner-Centered, Behaviorally Focused (NLB) framework structures the intervention cycle (diagnosis → co-design → implementation → feedback). The participatory cycle is hypothesized to operate through three mediating processes: (1) increased ownership and shared responsibility, (2) environmental alignment between portion size, food accessibility, and student preferences, and (3) norm formation and behavioral salience. These mechanisms are expected to reduce high-intensity plate waste and extreme leftovers. The magnitude of effects may be moderated by contextual factors, including the catering model, lunch organization, staffing constraints, structural feasibility, and baseline waste patterns.

The SDW program applied the NLB model [7] as a structured participatory implementation framework integrating needs assessment, stakeholder co-design, and behaviorally oriented environmental change. While participatory and environmental strategies have been previously documented [5,10,11,12,13,14,15,16], the present study provides real-world evidence that embedding these elements within an iterative NLB-informed cycle may contribute to meaningful reductions in plate waste, particularly in reducing high-intensity waste categories. These findings position SDW not only as a program evaluation but as a theory-informed implementation model for school-based sustainability interventions. The conceptual structure of this mechanism is illustrated in Figure 3. This model should be understood as a theory-informed interpretative framework derived from the present findings rather than as a formally tested structural model.

Based on the observed patterns, several theory-informed propositions can be formulated for future empirical testing:

**Proposition** **1.** *Participatory co-design processes increase perceived ownership and shared responsibility within institutional food environments, which in turn reduce high-intensity waste behaviors.*

**Proposition** **2.** *Environmental restructuring mechanisms (e.g., flexible portioning, self-service elements) reduce mismatch between appetite, time constraints, and portion size, leading primarily to reductions in extreme leftover categories rather than uniform increases in complete consumption.*

**Proposition** **3.** *Behavioral feedback loops created through direct engagement in monitoring processes enhance behavioral salience and norm formation within school settings.*

**Proposition** **4.** *Implementation fidelity measured quantitatively may not independently predict intervention outcomes; instead, contextual alignment and strategic coherence moderate effectiveness.*

These propositions require future testing using controlled or multilevel research designs. The present study should therefore be understood as generating mechanism-informed hypotheses rather than confirming causal theory.

### 4.5. Contextual and Organizational Barriers

Despite the overall improvement, food waste was not reduced in all schools. Qualitative observations indicated that factors such as long queues for lunch, limited dining space, and insufficient organization of supervision contributed to time pressure and the frequent disposal of partially eaten meals. These barriers are consistent with previous findings showing that time constraints and inadequate eating conditions are significant predictors of food waste in institutional settings.

Wansink et al. [17] demonstrated that shorter lunch breaks are associated with significantly lower consumption of fruits and vegetables. Similarly, Cohen et al. [18] showed that students require at least 25 min of effective seated time to meaningfully reduce plate waste. Cohen et al. [19] further emphasized that the organization of space and the scheduling of lunch periods play a critical role in determining how much food is actually consumed versus discarded.

These findings help explain the substantial variability in intervention outcomes observed across schools in the present study. In settings where organizational and environmental barriers remained unchanged, educational and participatory activities alone were insufficient to produce measurable reductions in food waste. This suggests that structural conditions of meal consumption may act as moderators of intervention effectiveness, limiting the impact of behavioral and educational components when not addressed simultaneously.

### 4.6. Educational Value and Student Engagement

An important feature of the SDW intervention was the active involvement of students not merely as recipients of educational content but as direct participants in the assessment and monitoring of food waste. By engaging students in the visual evaluation of plate waste at baseline (T1) and follow-up (T4), the measurement process itself became an embedded educational mechanism rather than a neutral data collection procedure.

This approach introduced a behavioral feedback loop: students were exposed in real time to the magnitude of uneaten food within their own environment. Such direct observation likely enhanced salience, personal accountability, and reflection on eating behaviors, extending beyond abstract knowledge acquisition.

Unlike conventional school-based programs in which students remain passive recipients of nutritional information, the SDW model positioned them as observers and contributors within the implementation process. Although student engagement has been widely recognized as important in nutrition education [10,16], relatively few interventions have incorporated learners into outcome monitoring itself.

The present findings suggest that embedding participatory measurement within intervention design may function simultaneously as an evaluative and educational mechanism, potentially strengthening the internalization of pro-environmental and pro-health norms, particularly in contexts where structural modifications are limited.

### 4.7. Strengths and Limitations of the Study

This study has several important strengths. First, it represents one of the few large-scale quasi-experimental studies on food waste reduction conducted in primary schools in Central and Eastern Europe. The inclusion of 37 public primary schools from all 18 districts of Warsaw ensured substantial diversity in organizational conditions and socio-economic context.

Second, the intervention was grounded in a solid theoretical framework—the NLB (Needs-Based, Learner-Centered, Behaviorally Focused) model [7], which integrates community needs assessment, behavioral focus, and active participation. This distinguishes the project from many previous initiatives that focused solely on education or environmental changes without incorporating behavioral and participatory components.

Third, the study adopted a multi-stakeholder approach, engaging school management, teachers, students, parents, and canteen staff. The “roundtable” method played a key role in fostering dialog, shared responsibility, and collaborative problem-solving. Consistent with the literature, such participatory processes enhance the sustainability of outcomes and promote a sense of ownership within the school community.

Finally, the intervention was implemented within the routine functioning of schools without requiring additional financial resources. This demonstrates that low-cost strategies based on participation and behavioral economics can produce measurable effects and can be feasibly integrated into municipal educational and environmental policies.

Several limitations of the study should be acknowledged. The study employed a quasi-experimental pre-post design without a control group, which introduces potential threats to internal validity, including historical effects, maturation processes occurring over the school year, and possible measurement reactivity associated with repeated visual assessment. However, both measurements were conducted within the same school year under stable municipal catering regulations, which may have partially reduced the likelihood of major structural historical confounders. In addition, the difference in the number of assessed meals between baseline (T1) and follow-up (T4) reflects routine fluctuations in daily attendance and meal uptake rather than structural sampling changes. Because analyses were conducted at the school level using aggregated mean scores and proportional distributions, minor differences in absolute meal counts are unlikely to have materially influenced the direction of observed effects. Nevertheless, this discrepancy should be interpreted as a limitation of field-based data collection. Participation was voluntary, which also raises the possibility of self-selection bias; schools opting into the program may have had higher baseline motivation, stronger administrative engagement, or greater openness to organizational change than non-participating institutions.

Given the absence of randomization and a comparison group, the observed changes cannot be interpreted as definitive causal effects of the intervention. Rather, they should be understood as indicative patterns of change occurring within a real-world municipal implementation context. The aim of the study was to assess feasibility and real-world change processes under organizational constraints typical of school-based sustainability interventions, aligning the project with an implementation-oriented research tradition rather than with causal efficacy testing.

A key limitation concerns the method used to assess plate waste. The visual assessment of uneaten food, conducted under the supervision of school coordinators and project trainers, facilitated student engagement and had clear educational value; however, it did not provide the level of precision achievable with weight-based measurements. Previous research indicates that visual methods are useful in large populations and educational contexts but may lead to underestimation of the actual amount of wasted food [20]. In contrast, weight-based methods are more reliable and allow for detailed analysis of both the quantity and type of food waste, but they are time-consuming and require additional organizational resources [21]. Future studies should consider combining visual assessment with quantitative measurement to enhance data reliability while preserving participant engagement, as recommended by systematic reviews on food waste monitoring in institutional settings [22].

The statistical analyses relied primarily on non-parametric paired comparisons and exploratory correlations. Multivariate modeling incorporating contextual covariates was not conducted due to the limited number of intervention units (*n* = 37 schools). Future studies with larger samples should apply multilevel or regression-based approaches to more precisely examine contextual moderators and clustering effects.

The results may have been influenced by external factors such as seasonal menu variations, parental expectations, or parallel health-promotion initiatives taking place in schools. Despite these limitations, the intervention demonstrates that positioning students as co-creators rather than passive recipients of change supports the internalization of pro-environmental attitudes and active participation in food waste reduction processes.

### 4.8. Practical Implications and Future Research

The findings of this study have important practical implications for schools, municipalities, and policymakers responsible for school catering systems. The results demonstrate that meaningful reductions in food waste can be achieved through low-cost, participatory, and behaviorally informed strategies without requiring substantial financial investment or structural reforms. Simple organizational changes—such as flexible portioning, the introduction of self-service elements, improved dining conditions, and the active involvement of students in decision-making—can be realistically implemented within the daily functioning of schools.

Importantly, the study shows that food waste reduction should not be approached solely as a nutritional or environmental issue but as a combined organizational, behavioral, and educational challenge. Interventions that integrate these dimensions may be more effective and sustainable than isolated educational campaigns or environmental modifications.

For policymakers and municipal authorities, structural adjustments within catering systems (e.g., portion flexibility, self-service elements, scheduling modifications) appear particularly relevant. The exploratory regression analysis (Table 4) further suggests that structural elements such as salad bar availability may facilitate greater reductions in high-intensity waste categories. This finding supports the prioritization of context-sensitive environmental adjustments within municipal school catering policies.

For school administrators, participatory roundtable processes may serve as a low-cost mechanism to enhance contextual fit and stakeholder ownership. For catering providers, alignment between portion size and student preferences may be more influential than informational campaigns alone.

Although the present findings suggest that participatory and organizational strategies may contribute to reductions in plate waste, these implications should be interpreted within the methodological boundaries of a quasi-experimental design without a control group. The observed changes cannot be attributed exclusively to the intervention, and therefore the program should not be interpreted as a universally effective model independent of context.

Scalability at the municipal level would require several enabling conditions, including: (i) institutional support from educational authorities, (ii) trained facilitators capable of moderating participatory processes, (iii) structural feasibility within existing catering systems, and (iv) sufficient organizational flexibility at the school level. The effectiveness of broader implementation is therefore likely to depend on contextual alignment rather than simple replication of intervention components.

Repeated exposure to waste monitoring may itself have functioned as a behavioral intervention component, introducing potential measurement reactivity.

Future research should focus on long-term follow-up to assess the sustainability of observed changes over time. Studies combining visual assessment with quantitative measurement of food waste are needed to enhance precision and allow for more detailed analysis of the types of food discarded. Additionally, randomized or controlled designs could help to isolate the specific contribution of participatory and behavioral components. Further investigation is also warranted into how contextual factors—such as lunch break duration, dining space organization, and catering models—moderate the effectiveness of school-based food waste interventions.

## 5. Conclusions

The present findings suggest that a structured, NLB-informed participatory implementation model may contribute to reducing plate waste in primary school settings. In approximately two-thirds of the schools, a tendency toward decreased levels of food waste was observed, suggesting that an approach based on community involvement and adaptation of activities to local conditions may facilitate favorable outcomes.

The findings further suggest that a greater potential for food waste reduction is associated with the simultaneous implementation of educational and organizational components. Schools that introduced actions combining these two elements appeared to achieve more positive changes, although these differences were not statistically significant. It can be assumed that the effectiveness of the intervention depends not only on the number of implemented actions but also on their coherence and the degree of staff engagement.

From a practical perspective, the program may serve as a context-sensitive reference framework for schools operating under comparable institutional conditions, while further controlled research is required to establish generalizable efficacy. Future research should consider combining visual and weight-based methods for assessing plate waste to improve measurement precision, as well as conducting longer-term follow-up observations to evaluate the sustainability of the observed effects over time.

## Figures and Tables

**Table 1:** Baseline Comparability of Schools Completing vs. Withdrawing from the Study.

Variable	Completed (*n* = 37)	Withdrew (*n* = 13)	Test	*p*-Value
Catering type (external/in-house)	22/15	8/5	χ^2^(1) = 0.15	0.700
Number of lunch breaks (2/3/4)	22/12/3	6/5/2	χ^2^(2) = 0.50	0.779
Advance menu availability (Yes/No)	36/1	13/0	χ^2^(2) = 0.81	0.667
Portion size choice (Yes/No)	4/33	0/13	Fisher	0.566
Meals served by staff (Yes/No)	37/0	13/0	χ^2^(2) = 0.81	0.667
Salad bar availability (Yes/No)	6/31	3/10	χ^2^(1) = 0.16	0.694
% fully consumed meals (T1)	38.6 (23.3–49.1 *)	31.8 (25.2–48.3 *)	Mann–Whitney U = 243	0.846
% fully uneaten meals (T1)	61.4 (50.9–76.7 *)	68.2 (51.7–74.8 *)	Mann–Whitney U = 243	0.846

* Values reported as median (Q1–Q3).

**Table 2:** Organization of School Catering in Participating Primary Schools at Baseline (T1) (*n* = 50).

Variable	Category	*n*	%
Type of catering organization	In-house kitchen	20	40
	External catering provider	30	60
Number of lunch breaks	2	26	52
	3	16	32
	4	5	16
Students and parents have prior access to weekly menu	Yes	50	100
	No	–	–
Different portion sizes available (larger/smaller portions)	Yes	10	20
	No	40	80
Meals served by canteen staff (no self-service)	Yes	50	100
	No	–	–
Salad bar available	Yes	9	18
	No	41	82
Students allowed to take additional servings	Yes	22	44
	No	28	56

**Table 3:** Comparison of Meal Consumption Categories between Baseline (T1) and Post-Intervention (T4) Measurements—Wilcoxon Signed-Rank Test.

Comparison Category (T4 − T1)	Z Statistic	*p*	Effect Size (r)	Direction	Interpretation
Number of assessed meals	−2.813	0.005	−0.46	↓	Slightly fewer meals were assessed at T4.
Meal fully consumed	−0.778	0.436	—	—	No statistically significant change.
¼ of portion left uneaten	−0.606	0.544	—	—	No statistically significant change.
½ of portion left uneaten	−3.164	0.002	−0.52	↓	Significant reduction in meals where only half of the portion was consumed.
¾ of portion left uneaten	−3.397	<0.001	−0.56	↓	Significant reduction in meals where only one-quarter of the portion was consumed.
Entire meal left uneaten	−4.052	<0.001	−0.67	↓	Significant reduction in completely uneaten meals.
Total number of uneaten meals	−2.867	0.004	−0.47	↓	Significant reduction in the overall number of uneaten meals.
% of meals fully consumed	−1.456	0.145	—	—	No statistically significant change.
% of meals uneaten	−1.430	0.153	—	—	No statistically significant change.

Legend: Wilcoxon signed-rank test (r = Z/√N; *n* = 37 schools). Values of *p* < 0.05 were considered statistically significant. Arrows (↓) indicate the direction of change between the baseline measurement (T1) and the post-intervention measurement (T4).

**Table 4:** School-level regression model predicting change in uneaten meals (T4 − T1).

Predictor	B (SE)	β	t	*p*	VIF
Intercept	−15.666 (10.877)	—	−1.440	0.160	—
Baseline % uneaten (T1)	0.560 (0.130)	0.595	4.321	<0.001	1.112
Catering type (in-house vs. external)	−7.659 (4.724)	−0.244	−1.621	0.116	1.327
Number of lunch breaks (≥3 vs. 2)	−3.996 (4.387)	−0.128	−0.911	0.370	1.163
Salad bar availability (yes vs. no)	−14.899 (6.088)	−0.359	−2.447	0.021	1.259
Ability to request additional portions (yes vs. no)	0.409 (4.553)	0.013	0.090	0.929	1.232
Portion size choice at ordering (yes vs. no)	−8.462 (7.445)	−0.172	−1.137	0.265	1.339

Dependent variable: change in the percentage of uneaten meals (T4 − T1). Negative B values indicate a greater reduction in plate waste. Binary variables were coded 0/1 (0 = reference category). Variance inflation factors indicated no multicollinearity concerns.

**Table 5:** Spearman’s rank correlations between the number of planned and implemented actions, implementation fidelity, and change in food waste levels (Δ waste = T4 − T1).

Variable 1	Variable 2	Spearman’s ρ	*p*-Value
Planned actions	Δ waste	−0.07	0.695
Implemented actions	Δ waste	0.07	0.662
Fidelity (%)	Δ waste	0.20	0.235

Note. ρ—Spearman’s rank correlation coefficient; *p*—two-tailed significance level. No statistically significant correlations were observed (all *p* > 0.05).

## Data Availability

The data supporting the findings of this study are available from the corresponding author upon reasonable request. The data are not publicly available due to institutional and administrative restrictions related to participating schools. The dataset does not contain personal or identifiable information.

## Supplementary Materials

The following supporting information can be downloaded at: https://www.mdpi.com/article/10.3390/nu18060885/s1. Supplementary Material S1: Structured Scenario of the Roundtable Meeting (T2) in the Schools Don’t Waste (SDW) Intervention.

## Abbreviations

The following abbreviations are used in this manuscript:

SDW	Schools Don’t Waste
NLB	Needs-Based, Learner-Centered, Behaviorally Focused approach
T1	Baseline measurement (pre-intervention)
T2	Roundtable meeting stage
T3	Implementation stage of educational and organizational actions
T4	Post-intervention evaluation
GWP	Global Warming Potential
SDGs	Sustainable Development Goals
IQR	Interquartile Range
