# Supplementary material for: The ‘Schools Don’t Waste’ Program: A Theory-Informed Participatory Intervention to Reduce Plate Waste in Public School Canteens
Source: Nutrients. 2026 Mar 11;18(6):885. doi: 10.3390/nu18060885 (PMC13029184; doi:10.3390/nu18060885)
Supplement: Supplementary file 1 [file nutrients-18-00885-s001.zip › nutrients-4171599-supplementary.pdf]

nutrients

MDPI

Supplementary Material S1

Structured Scenario of the Roundtable Meeting (T2) in the Schools Don't Waste (SDW) Intervention

Total duration: ~60 minutes

Facilitator: SDW project trainer

Participants: students, teachers, school coordinator, kitchen/canteen staff, school management, parents (if available)

1. Introduction and Objectives (10 minutes)

Facilitator tasks:

- Welcome participants and explain the purpose of the meeting.
- Present the main objective: reducing food waste in the school canteen.
- Emphasize that all participants have equal voices regardless of their role.

Key message to participants:

"Today, we will jointly analyze how food is wasted in our school and decide what we can realistically change together."

2. Presentation of Baseline Results (T1) (10 minutes)

Facilitator tasks:

- Present visual results from the plate waste assessment conducted in the school.
- Show proportions of plates: fully eaten, partially eaten, uneaten.
- Avoid blaming language; focus on facts and observations.

Guiding questions:

- What surprises you in these results?
- Did you expect this level of food waste?

3. Identification of Causes (15 minutes)

Participants discuss possible reasons for plate waste.

Facilitator writes all ideas visibly (board/flipchart).

Guiding questions:

- Why do students leave food on their plates?
- Are portion sizes adequate?
- Is there enough time to eat?
- Are meals attractive and tasty?
- Does the canteen environment support comfortable eating?

4. Generating Solutions (15 minutes)

Participants propose practical changes.

Rules:

- No idea is rejected at this stage.

- Focus on solutions that are feasible within the school.

Prompting areas if discussion slows:

- Portion sizes
- Choice and flexibility
- Organization of lunch breaks
- Canteen atmosphere
- Educational activities
- Student involvement

5. Selection of Priority Actions (5 minutes)

Group collectively selects:

- 1–2 main goals (e.g., “increase fully eaten meals by 10%”)
- 3–6 specific actions to implement

Each action must answer:

- What will be done?
- Who is responsible?
- When will it start?

6. Closing and Commitment (5 minutes)

Facilitator summarizes:

- Agreed goals
- Planned actions
- Roles of participants

Key closing message:

“This is our shared plan. Its success depends on all of us.”
